# Changes in serum BDNF levels associated with moderate-intensity exercise in healthy young Japanese men

**DOI:** 10.1186/2193-1801-2-678

**Published:** 2013-12-18

**Authors:** Akio Goda, Shohei Ohgi, Kazuhiro Kinpara, Kenta Shigemori, Kanji Fukuda, Eric B Schneider

**Affiliations:** Graduate school of Rehabilitation Science, Seirei Christopher University, 3453, Mikatahara-Cho, Hamamatsu-City, Shizuoka, 433-8558 Japan; Department of healthcare, Kansai University of Health and welfare science, 3-11-1, Asahigaoka, Kashiwara-City, Osaka, 582-0026 Japan; Department of Rehabilitation, Kinki University School of Medicine, 377-2 Ohno-higashi, Osaka-Sayama, Osaka, 589-8511 Japan; Johns Hopkins School of Medicine, Center for Surgical Trials and Outcomes Research, 600 N. Wolfe Street, Baltimore, MD 21205 USA

**Keywords:** Brain-derived neurotrophic factor, Japanese, Human, Physical activity, Aerobic exercise

## Abstract

**Purpose:**

The purpose of this study was to examine the influence of acute moderate-intensity exercise on serum brain-derived neurotrophic factor (BDNF) levels in healthy young Japanese men. BDNF is one of a family of neurotrophic factors involved in neuronal transmission, modulation and plasticity. Previous human-based studies have demonstrated that acute exercise leads to increases in BDNF; however, to date there has been no study conducted among Japanese male subjects.

**Methods:**

Forty young adult Japanese men (aged 24.1 ± 2.9 years) – reduced to a total of thirty three following subjection to exclusion criteria – performed 30 minutes of exercise at 60% of VO_2max_ on a stationary bicycle. Serum BDNF was analyzed both before and after exercise.

**Results:**

Eighteen of the total thirty three subjects demonstrated an increase in serum BDNF after exercise. However, on aggregate, the change in serum BDNF associated with exercise was not significant (p = .17).

**Conclusions:**

This is the first study to demonstrate that serum BDNF levels are not consistently increased by acute moderate-intensity exercise in Japanese men. It is likely that something in the lifestyle and/or environment of male Japanese subjects underlies the difference between our findings and studies conducted in other countries.

## Background

Approximately 35.6 million people worldwide were living with dementia in 2010, with the prevalence expected to increase to 65.7 million in 2030 and 115.4 million in 2050 (Prince et al. 2013). Japan is considered to be one of the most rapidly aging nations in the world. It is estimated that the number of Japanese people with dementia will increase from 1.56 million in 2000 to over 3 million by 2025 (Cabinet Office, Government of Japan 2012).

Numerous studies suggest that regular physical activity could reduce the incidence of cognitive impairment and dementia in older people at risk for these disorders. For example, Laurin et al. (2001) demonstrated significantly lower odds of developing cognitive impairment and Alzheimer’s disease (AD) in subjects with higher levels of physical activity suggesting that regular physical activity could represent an important and potent protective factor against cognitive decline and dementia in elderly persons. A recent meta-analysis of 29 studies involving aerobic exercise interventions reported modest but significant improvements in attention and processing speed, executive function and memory in exercise-trained subjects (Smith et al. 2010). Also, both cross-sectional (Erickson et al. 2009) and prospective (Erickson et al. 2011) human investigations suggest that higher aerobic fitness level is associated with larger hippocampal volume and improved neuronal health. These reports further suggest that the improvements in cognitive function associated with aerobic activity may be mediated by neurophysiological and structural changes in the brain (Erickson et al. 2009); however, the physiological mechanisms underlying these effects remain unclear.

One possible mechanism involves changes in brain derived neurotrophic factor (BDNF) levels associated with aerobic exercise. This relationship was first observed in animal studies which demonstrated that physical activity increases the expression of BDNF in the rat brain (Neeper et al. 1995). Since this discovery, a number of studies have sought to establish the link between the neurothrophin BDNF and post-exercise enhancement of mood and cognitive functions in humans (Zoladz and Pilc 2010). BDNF is an important molecular mediator of structural and functional plasticity in the brain and plays many important roles in nervous system function, including neuroplasticity, neuronal growth, repair and differentiation. Altered BDNF levels have been described in several neurological and psychiatric disorders including AD (Schindowski et al. 2008), Huntington’s disease (Ciammola et al. 2007), major depression (Laske et al. 2007) and schizophrenia (Reis et al. 2008). Furthermore, higher BDNF serum levels are associated with a slower rate of cognitive decline in AD patients (Laske et al. 2011).

Several studies in human subjects have demonstrated that acute aerobic exercise induced increased BDNF in peripheral blood (Nofuji et al. 2012; Schmolesky et al. 2013; Gustafsson et al. 2009; Rasmussen et al. 2009; Ferris et al. 2007; Gold et al. 2003; Rojas Vega et al. 2006; Tang et al. 2008). The acute effect of exercise on human serum BDNF levels is characterized as a transient, moderate (approximately 20 to 40%) increase (Gold et al. 2003; Rojas Vega et al. 2006; Tang et al. 2008). Serum BDNF levels rise during aerobic exercise, and quickly return to baseline levels upon exercise cessation, approximately 10–15 minutes after exercise termination (Rojas Vega et al. 2006; Tang et al. 2008). Ferris et al. (2007) suggested that low intensity exercise was insufficient to elevate BDNF levels relative to baseline, while high intensity exercise for a comparable duration significantly elevated serum BDNF levels.

While many studies in this field have been done in Western subject, there was a single study in Japanese subject (Nofuji et al. 2012). Nofuji et al. (2012) demonstrated an effect of acute aerobic exercise on peripheral blood BDNF levels in Japanese women. However, there has been no study to date that has investigated the effects of acute aerobic exercise on peripheral blood BDNF levels in Japanese men. Therefore, the aim of this study was to clarify the effect of acute physical activity on the circulating BDNF responses in Japanese men. In the present study, we measured the serum BDNF concentrations before and after aerobic exercise healthy young Japanese men subjects.

## Results

### GXT

The mean peak values of work rate (190.43 ± 29.06 W), VO_2max_ (37.19 ± 6.47 mL/min/kg), HR (173.73 ± 13.45 bpm), RER (1.29 ± 0.14), and Borg Scale (17.85 ± 1.78), during graded exercise test are presented in Table 1. These data are consistent with the ACSM criteria for estimating VO_2max_.

**Table 1:** **Physiological maximal values during graded exercise test (GXT)**

	Peak value
VO_2max_ (mL/min/kg)	37.2 (6.5)
Work rate (W)	190.4 (29.1)
HR (bpm)	173.7 (13.5)
RER	1.29 (0.14)
Borg Scale	17.9 (1.8)

Values shown are means (±SD). [N = 40].

*VO*_*2max*_ maximal oxygen consumption, *HR* heart rate, *RER* respiratory exchange ratio.

### Endurance ride measures

On the basis of the exclusion criteria, 7 subjects were excluded from the analysis (2 subjects with an over-load and 5 subjects with an under-load). An analysis was performed on 33 remaining subjects. The average of the physiological values collected during exercise (3 minutes rest, 3 minutes warm-up, and each 5 minutes average during 30 minutes of endurance ride) are shown in Table 2. These results suggest that exercise intensity was maintained at a moderate level during the endurance ride.

**Table 2:** **Change physiological values during the endurance rides**

	Rest	Warm-up	Ex1-5	Ex6-10	Ex11-15	Ex16-20	Ex21-25	Ex26-30
VO2 (mL/min/kg)	7.1 (2.4)	12.9 (2.1)	18.3 (2.7)	21.4 (3.2)	21.8 (3.5)	21.8 (3.5)	21.8 (3.6)	21.8 (3.5)
%VO2max (%)	20.4 (7.0)	36.0 (4.9)	51.3 (6.0)	59.5 (4.1)	60.6 (3.5)	60.5 (3.5)	60.4 (3.2)	60.4 (3.4)
W/kg	-	0.99 (0.17)	1.4 (0.21)	1.53 (0.27)	1.51 (0.28)	1.47 (0.29)	1.45 (0.28)	1.43 (0.27)
HR (bpm)	75.4 (11.3)	111.0 (12.3)	132.7 (15.1)	145.9 (13.8)	151.5 (14.1)	154.2 (13.4)	156.1 (13.8)	157.8 (12.8)
%HRR (%)^a^	-	32.3 (8.2)	52.1 (11.4)	64.0 (10.9)	69.1 (11.5)	71.6 (10.8)	73.3 (11.1)	74.8 (10.3)
RER	0.8 (0.07)	0.88 (0.08)	1.06 (0.11)	1.05 (0.1)	1.03 (0.11)	1.01 (0.11)	1.0 (0.09)	0.99 (0.09)
Borg scale	-	8.1 (1.8)	11.4 (2.5)	13.2 (2.2)	14.1 (2.1)	14.8 (2.1)	15.2 (2.0)	15.5 (2.2)

Values shown are means (±SD). [N = 33].

*VO*_*2*_ oxygen consumption, *VO*_*2max*_ maximal oxygen consumption, *HR* heart rate, *HRR* heart rate reserve, *RER* respiratory exchange ratio.

^a^(HR – rest HR)/(220 – age – rest HR) *100.

### Serum BDNF responses

Basal BDNF values, post-exercise BDNF values, pre-post change in serum BDNF (⊿BDNF) and pre-post percentage change in serum BDNF (%⊿BDNF) are presented in Table 3. 18 of 33 subjects demonstrated increased serum BDNF after exercise compared with their personal baseline. However, on average for the entire group, post-exercise serum BDNF values did not increase relative to the baseline average (p = 0.168). There is a positive correlation between basal BDNF value and post-exercise BDNF value (r = .475, p < 0.01). There are no significant differences between the group with increased BDNF and the one with decreased BDNF in baseline values (Table 4). Equally, there are no significant correlations between exercise-induced BDNF change and basal baseline values (Table 5).

**Table 3:** **BDNF values and change**

	Mean (SD)	Max value	Minimal value
Basal BDNF	14.9 (5.0)	26.1	3.2
Post-exercise BDNF	15.9 (5.6)	25.7	4.8
⊿BDNF^a^	1.0 (4.2)	10.57	-6
%⊿BDNF^b^	15 (59.9)	295.3	-45.6

[N = 33].

^a^post BDNF value - basal BDNF value.

^b^(post BDNF value - basal BDNF value)/basal BDNF value.

**Table 4:** **Results of** ***t*** **-test on basal values by group**

	Increased BDNF group	Decreased BDNF group	***p*** value
	[N = 18]	[N = 15]	
Basal BDNF (ng/ml)	14.2 (4.7)	15.8 (5.4)	0.40
Age (years)	23.2 (1.7)	24.8 (3.1)	0.11
Height (cm)	171.5 (6.4)	168.5 (7.8)	0.25
Weight (kg)	64.6 (10.8)	61.6 (8.6)	0.40
BMI	21.9 (2.8)	21.6 (2.1)	0.78
VO2max (ml/min/kg)	35.1 (5.6)	37.5 (7.6)	0.30
Peak value of GXT (W/kg)	2.9 (0.3)	3.16 (0.7)	0.20
Resting HR (bpm)	74.5 (9.9)	76.6 (13.2)	0.61
HR durind endurance exercise (bpm)	150.6 (15.3)	148.4 (10.2)	0.65

Values shown are mean (±SD). [N = 33].

*VO*_*2max*_ maximal oxygen consumption, *BMI* body mass index, *HR* heart rate.

**Table 5:** **The results of a correlation analysis between basal characteristics and BDNF change**

	⊿BDNF		%⊿BDNF	
	r	***p*** value	r	***p*** value
Age (years)	-0.25	0.15	-0.15	0.40
Height (cm)	-0.11	0.54	-0.14	0.41
Weight (kg)	0.00	0.99	-0.18	0.30
BMI	-0.05	0.77	-0.19	0.28
VO2max (ml/min/kg)	-0.06	0.72	0.06	0.74
Peak value of work rate (W/kg)	-0.02	0.92	0.08	0.65
Resting HR (bpm)	-0.13	0.45	-0.18	0.30

[N = 33].

*VO*_*2max*_ maximal oxygen consumption, *BMI* body mass index, *Resting HR* resting heart rate.

## Discussion

This is the first study to examine changes in serum BDNF levels associated with a single bout of aerobic exercise in healthy young Japanese men. Based upon previous research in non-Japanese populations, we hypothesized that moderate aerobic exercise would lead to significantly increased serum BDNF levels in Japanese male subjects. However, serum BDNF increase due to exercise was evident in only half of the subjects, and group mean values of serum BDNF were not significantly increased by exercise compared with the pre-exercise group mean. The study found that serum BDNF did not increase consistently in healthy young Japanese men exposed to 30 minutes of moderate exercise.

Several studies on non-Japanese populations have demonstrated that brief periods of moderate aerobic exercise are associated with transient increases of serum BDNF concentrations (Goekint et al. 2008; Gold et al. 2003; Gustafsson et al. 2009); however, a study by Castellano and White (2008) suggested a negative relationship between moderate aerobic exercise and serum BDNF. Nofuji et al. (2012) reported that healthy young Japanese women subjects did not display increased serum BDNF levels after moderate exercise. The results of the present study prove that healthy young Japanese male subjects did not exhibit increased serum BDNF levels. Moderate aerobic exercise therefore does not seem to affect serum BDNF levels in healthy young Japanese populations as a whole.

Basal BDNF values from other studies have been reported as 24.95 ± 7.28 ng/ml (Schmolesky et al. 2013) and 22.94 ± 9.12 ng/mL (Cho et al. 2012) in healthy young men. Nofuji et al. (2008) demonstrated basal BDNF levels of 23.63 ± 2.94 ng/ml in sedentary young Japanese men. Among participants in this study, baseline BDNF was 14.9 ± 5.0 ng/ml. Although slightly lower than the values reported in prior studies, this level can be considered to be within the normal range (1.5 to 30.9 ng/mL) for basal BDNF in healthy subjects (Knaepen et al. 2010).

One factor that might be associated with our findings is the possible effect of stress hormones. Schmolesky et al. (2013) showed that BDNF levels may actually decrease in sedentary control subjects (by approximately 13% on average), and suggested this may be due to stress related to the laboratory environment and/or blood collection. Thus, it is possible that an exercise induced increase in BDNF may be offset by a stress-induced decrease associated with the blood collection process. Other studies have shown that elevated stress, exogenous cortisol application, or glucocorticoid receptor agonism can lead to reduced BDNF levels (Pluchino et al. 2013). If stress levels are particularly high or individual stress reactions are particularly strong, they could obscure the impact of exercise on the BDNF level.

One other possible explanation for our findings is the effect of human BDNF gene polymorphism (Val66Met), which is more common among Japanese individuals than those of Western heritage. Shimizu et al. (2004) showed that there are significant differences between individuals from Japan (50.3%), Italy (43.2%) and the USA (27.1%) in genotype frequencies of BDNF 196G/A (Val66Met) polymorphism, with the prevalence being significantly higher in the Japanese population (p < 0.0001). Val66Met is known to impair intracellular trafficking and neuronal activity-dependent secretion of BDNF (Egan et al. 2003). It is thought that Val66Met polymorphism carriers demonstrate lower BDNF production during exercise compared with individuals who do not carry this polymorphism. Within the Japanese population, the prevalence of BDNF polymorphism is reported to be approximately 50% (Itoh et al. 2004; Itoh et al. 2005). Interestingly, this prevalence is approximately equal to the proportion of subjects demonstrating increased serum BDNF after exercise in the present study.

Although serum levels of BDNF can be affected by BDNF production in peripheral cells or endocrine organs, including vascular endothelial cells (Nakahashi et al. 2000), immune cells (e.g. T and B lymphocytes) (Besser and Wank 1999; Kerschensteiner et al. 1999) and submandibular glands (Tsukinoki et al. 2007; Tsukinoki et al. 2006), the brain is considered to be the major contributor to exercise-related increases in circulating BDNF. Rasmussen et al. (2009) reported that the brain contributes almost 75% of all BDNF in circulation under normal circumstances, and that the brain generally demonstrates a significant increase in BDNF production during prolonged exercise (a 2- to 3-fold increase of the production at rest). The BDNF polymorphism found in half of the Japanese population affects intracellular trafficking of BDNF, reducing neuronal activity-dependent BDNF release by approximately 25% (Chen et al. 2008; Egan et al. 2003). While we were unable to directly measure the BDNF polymorphism for each individual participant, we believe there is a possibility that the prevalence of BDNF polymorphism among Japanese individuals might account for our finding that, on average, BDNF levels do not increase after exercise.

Limitations of this study include the absence of control groups (both a rest control group and one consisting of subjects of Western heritage) or a vigorous exercise subgroup to explore possible dose–response relationships between exertion-level and BDNF levels, as well as the lack of measurements of gene polymorphism (Val66Met) among our Japanese male subjects. Further studies are needed to explain changes in serum BDNF levels associated with exercise, as well as to elucidate the effect of BDNF polymorphism on exercise-induced BDNF secretion in Japanese subjects. Future studies should ascertain BDNF polymorphism status for each study participant, and included in analyses of the relationship between exercise and serum BDNF levels. Also, the influence of different levels of exercise intensity, its duration and type should be compared across sexes, age groups, health conditions, and fitness levels in order for exercise-induced BDNF secretion among Japanese individuals to be fully understood.

In conclusion, an exposure to 30 minutes of moderate aerobic exercise was not found to be associated with a consistent increase in serum BDNF concentrations in healthy young Japanese men. It is likely that something in the lifestyle and/or environment of male Japanese subjects underlies the difference between our findings and studies conducted in other countries.

## Methods

### Subjects and study design

The study population consisted of 40 healthy young Japanese men (mean age 24.1 ± 2.9 year; mean height 170.6 ± 6.7 cm; mean weight 64.8 ±9.4 kg; mean BMI 22.2 ±2.4 kg/m^2^; mean ± SE) with a sedentary lifestyle, which was defined as not having engaged in 30 minutes or more of purposeful physical activity per day at least three times a week over the previous 6 months. All subjects denied the presence of cardiopulmonary, metabolic, and musculoskeletal disease. All experiments were conducted in accordance with the Declaration of Helsinki and approved by the institutional review board of Seirei Christopher University, Japan. Written informed consent was also obtained from all participants.

### Study protocol

The study was conducted in two phases. In the first phase, each subject performed a graded exercise test (GXT) on a stationary bicycle to determine the work load that corresponded to 60% maximal oxygen consumption (VO_2max_) for that individual. This level of exertion was chosen because previous studies on subjects from Western populations used this intensity (Castellano and White 2008; Gold et al. 2003; Schulz et al. 2004). During the second phase, blood samples were collected from the subjects immediately before and after 30 minute endurance rides with a fixed exercise intensity level of 60% VO_2max_. There was a rest period of 48 hours between the GXT and the endurance ride. Participants were instructed to forgo strenuous exercise for 24 hours prior to both the baseline VO_2max_ measurement and the 30 minute endurance ride. Similarly, food, caffeine, alcohol intake and smoking were prohibited during the 3 hours prior to each phase.

### Graded exercise test

Baseline VO_2max_ was evaluated using a cycle ergometer on which participants exercised to volitional fatigue. Cardiopulmonary and metabolic parameters, maximal work rate, VO_2max_, and respiratory exchange ratio (RER) were determined on a breath-by-breath basis with samples averaged for 5-second intervals using the Aero Monitor AE-310 s (Minato Ika, Japan). Heart rate (HR) and rhythm were monitored during the GXT via electrocardiography (Bedside monitor scope 1; Nihon Kohden, Japan). We used the American College of Sports Medicine (ACSM) criteria for reaching VO_2max_, in which a participant meets at least two of the following criteria: (1) a leveling off of the VO_2_; (2) a rate of perceived exertion >17, using Borg’s scale; (3) volitional exhaustion; and (4) achievement of the participant’s age-predicted maximal heart rate [calculated from (220 – age)]. We defined the VO_2max_ plateau as a VO_2_ change < 2 mL kg - 1 min - 1 over the last 60 seconds of the test.

### Endurance ride

After a 3 minutes’ warm-up period at a power of 50 w, each subject performed 30 minutes of exercise at a power output that corresponded to 60% of his VO_2max_, as determined using the GXT. The power output was adjusted in increments of 2 w to reach 60% of VO_2max_ ± 100 mL/min/kg, as needed. Metabolic, ventilator, and heart rate parameters were collected at 1-minute intervals during the rides.

### Blood sampling and analysis

Basal and post-exercise BDNF levels were examined by drawing a 5-mL blood sample from the antecubital vein, with the subject in a sitting position, into a vacuum blood collection tube containing blood separating agent (NP-SP1029, NIPRO, Osaka, Japan), and using 21 gauge needles. The blood was sampled within 5 minutes before exercise initiation and 3 minutes post-exercise. It was clotted at room temperature for one hour and then centrifuged (Tabletop Centrifuge 2040, Kubota, Tokyo, Japan) at 3000 g for 15 minutes and the supernatant was decanted and stored in a -20°C freezer until analysis (approximately 2 to 3 months). Serum BDNF levels were determined using a commercially available quantitative sandwich enzyme-linked immunosorbent assay (ELISA) kit, in accordance with protocols provided by the test manufacturer (R& D Systems, USA). Each sample was tested twice with a sensitivity threshold of 20 pg/mL, producing an intra-assay variance under 6.2%, which was within the range specified by the manufacturer.

### Exclusion criteria

An analysis was conducted excluding subjects with a longer than 5 minutes’ period of deviation from the appropriate load strength (outside the range of 50-70% of VO2max at average value for each minute) during the 30 minutes endurance ride.

### Statistical analysis

Paired t-tests were used to compare serum BDNF levels measured prior to exercise onset, with BDNF levels measured after completion of the endurance rides. The statistical significance level was set at p < 0.05, and data were presented as mean ± standard deviation (SD). All statistical analyses were conducted using the software package (IBM SPSS Statistics 19 for Windows, Chicago, IL).

## Abbreviations

AD: Alzheimer’s disease
BDNF: Brain-derived neurotrophic factor
BMI: Body mass index
VO2: Oxygen consumption
VO2max: Maximal oxygen consumption
GXT: Graded exercise tests
HR: Heart rate
HRR: Heart rate reserve
RER: Respiratory exchange ratio
SNP: Single nucleotide polymorphism
Val: Valine allele
Met: Methionine allele.

## References

[CR1] Besser M, Wank R (1999). Cutting edge: clonally restricted production of the neurotrophins brain-derived neurotrophic factor and neurotrophin-3 mRNA by human immune cells and Th1/Th2-polarized expression of their receptors. J Immunol.

[CR2] (2012). The number of elderly people with dementia.

[CR3] Castellano V, White LJ (2008). Serum brain-derived neurotrophic factor response to aerobic exercise in multiple sclerosis. J Neurol Sci.

[CR4] Chen Z-Y, Bath K, McEwen B, Hempstead B, Lee F (2008). Impact of genetic variant BDNF (Val66Met) on brain structure and function. Novartis Found Symp.

[CR5] Cho HC, Kim J, Kim S, Son YH, Lee N, Jung SH (2012). The concentrations of serum, plasma and platelet BDNF are all increased by treadmill VO_2_max performance in healthy college men. Neurosci Lett.

[CR6] Ciammola A, Sassone J, Cannella M, Calza S, Poletti B, Frati L, Squitieri F, Silani V (2007). Low brain-derived neurotrophic factor (BDNF) levels in serum of Huntington’s disease patients. Am J Med Genet B Neuropsychiatr Genet.

[CR7] Egan MF, Kojima M, Callicott JH, Goldberg TE, Kolachana BS, Bertolino A, Zaitsev E, Gold B, Goldman D, Dean M, Lu B, Weinberger DR (2003). The BDNF val66met polymorphism affects activity-dependent secretion of BDNF and human memory and hippocampal function. Cell.

[CR8] Erickson KI, Prakash RS, Voss MW, Chaddock L, Hu L, Morris KS, White SM, Wójcicki TR, McAuley E, Kramer AF (2009). Aerobic fitness is associated with hippocampal volume in elderly humans. Hippocampus.

[CR9] Erickson KI, Voss MW, Prakash RS, Basak C, Szabo A, Chaddock L, Kim JS, Heo S, Alves H, White SM, Wojcicki TR, Mailey E, Vieira VJ, Martin SA, Pence BD, Woods JA, McAuley E, Kramer AF (2011). Exercise training increases size of hippocampus and improves memory. Proc Natl Acad Sci U S A.

[CR10] Ferris LT, Williams JS, Shen C-L (2007). The effect of acute exercise on serum brain-derived neurotrophic factor levels and cognitive function. Med Sci Sports Exerc.

[CR11] Goekint M, Heyman E, Roelands B, Njemini R, Bautmans I, Mets T, Meeusen R (2008). No influence of noradrenaline manipulation on acute exercise-induced increase of brain-derived neurotrophic factor. Med Sci Sports Exerc.

[CR12] Gold SM, Schulz KH, Hartmann S, Mladek M, Lang UE, Hellweg R, Reer R, Braumann KM, Heesen C (2003). Basal serum levels and reactivity of nerve growth factor and brain-derived neurotrophic factor to standardized acute exercise in multiple sclerosis and controls. J Neuroimmunol.

[CR13] Gustafsson G, Lira CM, Johansson J, Wisén A, Wohlfart B, Ekman R, Westrin A (2009). The acute response of plasma brain-derived neurotrophic factor as a result of exercise in major depressive disorder. Psychiatry Res.

[CR14] Itoh K, Hashimoto K, Kumakiri C, Shimizu E, Iyo M (2004). Association between brain-derived neurotrophic factor 196 G/A polymorphism and personality traits in healthy subjects. Am J Med Genet B Neuropsychiatr Genet.

[CR15] Itoh K, Hashimoto K, Shimizu E, Sekine Y, Ozaki N, Inada T, Harano M, Iwata N, Komiyama T, Yamada M, Sora I, Nakata K, Ujike H, Iyo M (2005). Association study between brain-derived neurotrophic factor gene polymorphisms and methamphetamine abusers in Japan. Am J Med Genet B Neuropsychiatr Genet.

[CR16] Kerschensteiner M, Gallmeier E, Behrens L, Leal VV, Misgeld T, Klinkert WE, Kolbeck R, Hoppe E, Oropeza-Wekerle RL, Bartke I, Stadelmann C, Lassmann H, Wekerle H, Hohlfeld R (1999). Activated human T cells, B cells, and monocytes produce brain-derived neurotrophic factor in vitro and in inflammatory brain lesions: a neuroprotective role of inflammation?. J Exp Med.

[CR17] Knaepen K, Goekint M, Heyman EM, Meeusen R (2010). Neuroplasticity - exercise-induced response of peripheral brain-derived neurotrophic factor: a systematic review of experimental studies in human subjects. Sports Med.

[CR18] Laske C, Stransky E, Eschweiler GW, Klein R, Wittorf A, Leyhe T, Richartz E, Köhler N, Bartels M, Buchkremer G, Schott K (2007). Increased BDNF serum concentration in fibromyalgia with or without depression or antidepressants. J Psychiatr Res.

[CR19] Laske C, Stellos K, Hoffmann N, Stransky E, Straten G, Eschweiler GW, Leyhe T (2011). Higher BDNF serum levels predict slower cognitive decline in Alzheimer’s disease patients. Int J Neuropsychopharmacol.

[CR20] Laurin D, Verreault R, Lindsay J, MacPherson K, Rockwood K (2001). Physical activity and risk of cognitive impairment and dementia in elderly persons. Arch Neurol.

[CR21] Nakahashi T, Fujimura H, Altar CA, Li J, Kambayashi J, Tandon NN, Sun B (2000). Vascular endothelial cells synthesize and secrete brain-derived neurotrophic factor. FEBS Lett.

[CR22] Neeper SA, Gómez-Pinilla F, Choi J, Cotman C (1995). Exercise and brain neurotrophins. Nature.

[CR23] Nofuji Y, Suwa M, Moriyama Y, Nakano H, Ichimiya A, Nishichi R, Sasaki H, Radak Z, Kumagai S (2008). Decreased serum brain-derived neurotrophic factor in trained men. Neurosci Lett.

[CR24] Nofuji Y, Masataka S, Sasaki H, Ichimiya A, Nishichi R, Kumagi S (2012). Different circulating brain-derived neurotrophic factor responses to acute exercise between physically active and sedentary subjects. J Sports Sci Med.

[CR25] Pluchino N, Russo M, Santoro AN, Litta P, Cela V, Genazzani AR (2013). Sterioid hormones and BDNF. Neuroscience.

[CR26] Prince M, Bryce R, Albanese E, Wimo A, Ribeiro W, Ferri CP (2013). The global prevalence of dementia: a systematic review and metaanalysis. Alzheimers Dement.

[CR27] Rasmussen P, Brassard P, Adser H, Pedersen MV, Leick L, Hart E, Secher NH, Pedersen BK, Pilegaard H (2009). Evidence for a release of brain-derived neurotrophic factor from the brain during exercise. Exp Physiol.

[CR28] Reis HJ, Nicolato R, Barbosa IG, Prado PH T d, Romano-Silva MA, Teixeira AL (2008). Increased serum levels of brain-derived neurotrophic factor in chronic institutionalized patients with schizophrenia. Neurosci Lett.

[CR29] Rojas Vega S, Strüder HK, Vera Wahrmann B, Schmidt A, Bloch W, Hollmann W (2006). Acute BDNF and cortisol response to low intensity exercise and following ramp incremental exercise to exhaustion in humans. Brain Res.

[CR30] Schindowski K, Belarbi K, Buée L (2008). Neurotrophic factors in Alzheimer’s disease: role of axonal transport. Genes Brain Behav.

[CR31] Schmolesky MT, Webb DL, Hansen RA (2013). The effects of aerobic exercise intensity and duration on levels of brain-derived neurotrophic factor in healthy men. J Sports Sci Med.

[CR32] Schulz KH, Gold SM, Witte J, Bartsch K, Lang UE, Hellweg R, Reer R, Braumann KM, Heesen C (2004). Impact of aerobic training on immune-endocrine parameters, neurotrophic factors, quality of life and coordinative function in multiple sclerosis. J Neurol Sci.

[CR33] Shimizu E, Hashimoto K, Iyo M (2004). Ethnic difference of the BDNF 196G/A (val66met) polymorphism frequencies: the possibility to explain ethnic mental traits. Am J Med Genet B Neuropsychiatr Genet.

[CR34] Smith PJ, Blumenthal JA, Hoffman BM, Cooper H, Strauman TA, Welsh-Bohmer K, Browndyke JN, Sherwood A (2010). Aerobic exercise and neurocognitive performance: a meta-analytic review of randomized controlled trials. Psychosom Med.

[CR35] Tang SW, Chu E, Hui T, Helmeste D, Law C (2008). Influence of exercise on serum brain-derived neurotrophic factor concentrations in healthy human subjects. Neurosci Lett.

[CR36] Tsukinoki K, Saruta J, Sasaguri K, Miyoshi Y, Jinbu Y, Kusama M, Sato S, Watanabe Y (2006). Immobilization stress induces BDNF in rat submandibular glands. J Dent Res.

[CR37] Tsukinoki K, Saruta J, Muto N, Sasaguri K, Sato S, Tan-Ishii N, Watanabe Y (2007). Submandibular glands contribute to increases in plasma BDNF levels. J Dent Res.

[CR38] Zoladz JA, Pilc A (2010). The effect of physical activity on the brain derived neurotrophic factor: from animal to human studies. J Physiol Pharmacol.

